# Hemispheric Lateralization of Arithmetic Facts and Magnitude Processing for Two-Digit Numbers

**DOI:** 10.3389/fnhum.2020.00088

**Published:** 2020-03-24

**Authors:** Stefanie Jung, Korbinian Moeller, Hans-Otto Karnath, Elise Klein

**Affiliations:** ^1^Junior Research Group Neuro-Cognitive Plasticity, Leibniz-Institut für Wissensmedien, Tübingen, Germany; ^2^Research Methods and Mathematical Psychology, Eberhard Karls Universität Tübingen, Tübingen, Germany; ^3^LEAD Graduate School & Research Network, University of Tübingen, Tübingen, Germany; ^4^Center of Neurology, Section for Neuropsychology, Hertie Institute for Clinical Brain Research, University of Tübingen, Tübingen, Germany; ^5^CNRS UMR 8240, Laboratory for the Psychology of Child Development and Education, Paris, France

**Keywords:** interhemispheric communication, number comparison task, number bisection task, two-digit number processing, hemispheric lateralization

## Abstract

In the human brain, a (relative) functional asymmetry (i.e., laterality; functional and performance differences between the two cerebral hemispheres) exists for a variety of cognitive domains (e.g., language, visual-spatial processing, etc.). For numerical cognition, both bi-lateral and unilateral processing has been proposed with the retrieval of arithmetic facts postulated as being lateralized to the left hemisphere. In this study, we aimed at evaluating this claim by investigating whether processing of multiplicatively related triplets in a number bisection task (e.g., 12_16_20) in healthy participants (*n* = 23) shows a significant advantage when transmitted to the right hemisphere only as compared to transmission to the left hemisphere. As expected, a control task revealed that stimulus presentation to the left or both visual hemifields did not increase processing disadvantages of unit-decade incompatible number pairs in magnitude comparison. For the number bisection task, we replicated the multiplicativity effect. However, in contrast to the hypothesis deriving from the triple code model, we did not observe significant hemispheric processing asymmetries for multiplicative items. We suggest that participants resorted to keep number triplets in verbal working memory after perceiving them only very briefly for 150 ms. Rehearsal of the three numbers was probably slow and time-consuming so allowing for interhemispheric communication in the meantime. We suggest that an effect of lateralized presentation may only be expected for early effects when the task is sufficiently easy.

## Introduction

One of the most important postulates of the Triple Code Model (henceforth TCM) of numerical cognition is the distinction between the representation of number magnitude processing on the one hand and arithmetic facts and their verbally mediated retrieval from long term memory on the other hand (Dehaene and Cohen, 1995, 1997; Dehaene et al., 2003). As regards number magnitude processing, the TCM suggests a bilateral fronto-parietal network around the intraparietal sulcus (IPS) to be dedicated to the representation and mental manipulation of numerical quantities – for instance, when calculations need to be performed (e.g., 124–56). In contrast, tasks such as multiplication with small numbers (e.g., 3 × 2) are supposed to be solved by arithmetic fact retrieval subserved by a left-hemispheric network including perisylvian language areas as well as the angular gyrus (Dehaene et al., 2003). As such number magnitude is assumed to be represented redundantly in both hemispheres of the human brain, whereas the verbal representation of arithmetic facts is postulated for the left hemisphere of the human brain only.

The current view is that arithmetic facts are stored and retrieved in a verbal code (Dehaene et al., 2003). Neuro-functional evidence on the neural networks underlying verbally mediated fact retrieval stems primarily from studies that investigating the acquisition of arithmetic facts by means of drill trainings of difficult multiplication problems (e.g., 43 × 9 = ___; Bloechle et al., 2016; Delazer et al., 2003, 2005; Grabner et al., 2009; Ischebeck et al., 2006). Consistently, stronger activation was found in left-hemispheric perisylvian language areas as well as the left angular gyrus (e.g., Delazer et al., 2003, 2005; Ischebeck et al., 2006; Grabner et al., 2009) and the left hippocampus (e.g., Bloechle et al., 2016; Klein et al., 2019) for trained problems as opposed to untrained problems after the training. These activation patterns are assumed to reflect automatic verbally mediated retrieval of arithmetic facts from long-term memory (Delazer et al., 2003; Ischebeck et al., 2006; Bloechle et al., 2016).

In order to investigate the processing of arithmetic facts and number magnitude within one task, the number bisection task (NBT; Nuerk et al., 2002) was proposed. In the NBT, participants have to evaluate whether the central number of a triplet (e.g., 11_13_15) corresponds to the arithmetic integer mean of the interval defined by the two outer numbers. Triplets which are part of a multiplication table (21_24_27) provided a processing advantage as compared to non-multiplicative triplets (19_22_25, cf. Nuerk et al., 2002) by activating multiplication fact knowledge. Wood et al. (2008) replicated these findings and observed that processing of multiplicative triplets was specifically associated with activation in left-hemispheric perisylvian language areas and the angular gyrus (see also Klein et al., 2016 for a re-analysis). However, concurrent articulation led to relative slowing of processing multiplicative triplets in the NBT, which reduced the multiplicativity effect (Moeller et al., 2011).

These results support the central postulation of the TCM that arithmetic facts are processed in the left hemisphere only. This argument is primarily based on classical neuropsychological single-case studies on brain-lesioned patients. For instance, patient BOO (Dehaene and Cohen, 1997), patient WT (Zaunmuller et al., 2009) or patient VOL (Cohen and Dehaene, 2000), who suffered from left-hemispheric lesions, showed severe selective impairments in multiplication, which is solved by arithmetic fact retrieval. However, existing case studies of brain-lesioned patients do not support the assumption of the TCM that arithmetic facts are processed in a left-lateralized manner consistently: for instance, Granà et al. (2006) reported the case of patient PN who showed circumscribed deficits in multiplication following a right-hemispheric lesion. Moreover, Salillas and coworkers (Salillas et al., 2012) reported an association of multiplication performance and the right IPS by inducing a virtual lesion using transcranial magnetic stimulation.

In view of these inconsistent findings, the question whether the verbal representation of arithmetic facts is indeed lateralized to the left hemisphere is far from being answered comprehensively. Also, findings from various fMRI studies cannot provide sufficient evidence for isolated left-hemispheric activation for arithmetic fact retrieval as they often observed bilateral activation of perisylvian language areas (e.g., Dehaene et al., 1999; Stanescu-Cosson et al., 2000; Grabner et al., 2009; Klein et al., 2013a, b, 2016; Bloechle et al., 2016).

Therefore, it would be important to obtain converging evidence from healthy adult participants substantiating the theoretical claim that verbally mediated arithmetic fact retrieval is lateralized to the left hemisphere.

To this end, we used a task indicative of arithmetic fact retrieval in a divided visual field paradigm. In this divided visual field paradigm, respective stimuli are presented either unilaterally in the right or the left visual hemifield or bilaterally in both visual fields. When the stimuli are presented unilaterally into one visual hemifield only, visual input is initially only transmitted into the contralateral hemisphere. Evidence for the successful application of such divided visual field paradigms can be found in various domains (e.g., language: Geffen et al., 1971; Brysbaert, 1994; numerical cognition: Dimond and Beaumont, 1971; Hatta et al., 2002; Ratinckx et al., 2006; Hildebrandt et al., 2016). To give an example for the principle of this paradigm, when a stimulus is presented in the left visual hemifield, it would first be transferred to the right hemisphere; left-hemispheric processing of the respective stimuli would only occur after further transmission of the processed stimulus to the left hemisphere via interhemispheric transcallosal fibers. In case arithmetic facts are indeed processed exclusively in the left hemisphere, unilateral input into the left visual hemifield and thus initial transmission to the right hemisphere should lead to a processing disadvantage, reflected by, for instance, longer response latencies and lower accuracy compared to unilateral presentation of the stimuli into the right visual hemifield. Evidence for interhemispheric processing and its modulation has been provided by several studies using tDCS on the non-dominant hemisphere for the task at hand in higher cognitive processing, such as arithmetic fact retrieval (e.g., Clemens et al., 2013; for anodal stimulation) and primary perceptual processing (e.g., Bocci et al., 2018; for vision acuity).

Ratinckx et al. (2006) demonstrated that the divided visual field paradigm showed differential effects for the case of the unit-decade compatibility effect in two-digit number magnitude comparison, which is supposed to be processed in the right hemisphere (Wood et al., 2006; for a review see Nuerk et al., 2011). The unit-decade compatibility effect describes the finding that magnitude comparison in compatible number pairs (i.e., when separate comparisons of decade and unit digits lead to the same decision, e.g., in 42_57, 4 < 5 and 2 < 7) is easier than in incompatible number pairs (in which separate comparisons of decade and unit digits lead to opposing decisions, e.g., in 47_62; 4 < 6, but 7 > 2). In the study by Ratinckx et al. (2006), the disadvantage for the more demanding incompatible items was smaller when stimuli were presented unilaterally in the left visual hemifield or bilaterally to both visual fields and thus allowed initial right-hemispheric processing.

In the current study, we aimed at realizing a similar setting for the retrieval of arithmetic facts. To this end, we evaluated modulations of the multiplicativity effect in the NBT (Nuerk et al., 2002; Wood et al., 2008) in a divided visual field paradigm. In the NBT, the multiplicativity effect describes faster response times and lower error rates for triplets, which are part of a multiplication table (e.g., 21_24_27) as compared to number triplets which are not (e.g., 22_25_28, Nuerk et al., 2002; Moeller et al., 2009). Additionally, the multiplicativity effect was associated with activation of left-hemispheric language areas and the angular gyrus (Wood et al., 2008). It has been argued that multiplicativity of a triplet provides a processing advantage by activating multiplication fact knowledge (Nuerk et al., 2002).

For our divided visual field paradigm on the NBT, we used the stimulus set of Moeller et al. (2009). As a control task, we replicated the experiment by Ratinckx et al. (2006) on the unit-decade compatibility effect in magnitude comparison. To ensure that results are not confounded by stimulus specificities we created magnitude comparison stimuli only using numbers from the NBT stimulus set. This way, the magnitude comparison task served two purposes: on the one hand, it was used to verify that participants indeed could perceive and process the respective two-digit numbers which were presented only briefly at perifoveal positions. On the other hand, the task was used as a proof of concept: by replicating the results of Ratinckx et al. (2006) on modulation of the compatibility effect by lateralized presentation, we aimed at verifying that our experimental setting was valid.

In sum, the present study aimed at investigating whether the verbal representation of arithmetic facts is indeed lateralized to the left hemisphere of the human brain as put forward by the TCM (e.g., Dehaene and Cohen, 1995, 1997; Dehaene et al., 2003). Therefore, we investigated whether the processing of multiplicative triplets shows a significant advantage when stimuli are initially transmitted to the right hemisphere only. In particular, our hypotheses were as follows: as regards the number magnitude comparison control task, we expected to replicate the results of Ratinckx et al. (2006) of a modulation of the compatibility effect by lateralized presentation. In particular, presentation of number pairs in the left or bilaterally in both visual hemifields should reduce the disadvantage for incompatible number pairs in magnitude comparison. With respect to the NBT, we expected to replicate the multiplicativity effect. However, multiplicativity should only facilitate bisection performance when items were presented in the right visual hemifield or bilaterally in both visual fields because in this case input is directly transmitted to the left hemisphere of the brain for which the verbal representation of arithmetic facts is postulated.

## Materials and Methods

### Participants

Prior to data collection, we calculated the necessary sample size for the used within-participant design comparing effects of lateralized stimulus presentation for magnitude comparison and arithmetic fact retrieval in the number bisection task based on effect sizes reported in prior studies. For the multiplicativity effect in the NBT, both small (Cohen’s *d* = 0.2–0.4, Moeller et al., 2011) and large effect sizes (Cohen’s *d* = 0.8, Nuerk et al., 2002) were observed so far. For the effect of lateralization of presentation, a medium effect size was found the study by Ratinckx et al. (2006). Expecting a medium effect size of about *d* = 0.6 for both effects, a sample size of 21 participants should allow for detecting the respective effect with enough statistical power. In particular, we used the following parameters for the *a priori* sample calculation: As we used a repeated measure within-participant design, we considered one group of participants. For the effect size, we assumed a partial eta square of η^2^*_*p*_* = 0.20. We expected an alpha error probability of *p* = 0.05 and a power of 0.95. Furthermore, we compared three different measurements (i.e., bilateral, right lateralized and left-lateralized item presentation). Among these repeated measures, we expected a high correlation of 0.85.

In total, 32 right-handed healthy volunteers (7 male, mean age = 24.5 years; *SD* = 3.56), who graded ‘4’ or better in mathematics in their school-leaving certificate (with grades in Germany ranging from 1 to 6 with 1 being the grade), were recruited via public announcements. All participants had normal or corrected to normal vision and reported no history of neurological or psychiatric disorders. We excluded one participant from data analysis as she reported to have suffered from math anxiety during school.

Thus, data from 31 participants (6 male, mean age = 24.34 years; *SD* = 3.03), were considered for the analyses. 30 participants had more than 10 years of formal education. Eight participants are exposed to mathematics in their profession. The Edinburgh Handedness Inventory (Oldfield, 1971) was used to determine handedness. Participants were categorized as right−handed using the cut-off criterion of LQ > + 50, indicating them to fall in the first decile or higher of right-handedness. Eye dominance was also recorded for both distance (right = 21 participants, left = 10 participants) and proximity (right = 11 participants, left = 3 participants, not defined = 17 participants).

The study was approved by the local Ethics Committee (082/2018BO2) and was performed in compliance with the latest version of the Code of Ethics of the World Medical Association (Declaration of Helsinki). All participants gave their written informed consent prior to the study and received compensatory payment.

### Procedure

Data were collected in individual 2-h testing sessions. Within one session, two experimental tasks had to be completed: a number magnitude comparison task and a NBT. The order of both tasks was counterbalanced across participants to minimize order effects. These two experimental tasks were followed by a control task assessing multiplication fact retrieval. Task instructions emphasized both speed and accuracy in all tasks. Furthermore, the left and right control key on the keyboard were used as response buttons in all tasks. Stimulus presentation, response times and accuracy were recorded using Presentation software version 20.03 (Neurobehavioral Systems Inc., Albany, CA, United States).

### Task and Stimuli

Both in the experimental and the control task participants were required to give “Yes” or “No” responses by pressing either the left (i.e., “No” response: left Ctrl-key press) or the right response button (i.e., “Yes” response: right Ctrl-key press) with their left and right index finger, respectively. In all tasks, problems were presented in pseudo-randomized order, preventing a direct repetition of the same problem. Additionally, the sequence was manipulated such that no more than three correct or false trials, respectively, were presented in a row. This also applied to the side of presentation (i.e., left, right, bilateral) of items in both experimental tasks.

#### Number Bisection Task

In the NBT, 200 two-digit number triplets (100 correctly bisected: e.g., 18_24_30 and 100 incorrectly bisected: e.g., 17_18_30), covering the range from 11 to 99 were presented (cf. Moeller et al., 2009 for the same item set). The same item set was used for each condition (i.e., right, left, and bilateral), resulting in a total of 600 trials. At the beginning, twelve randomly chosen triplets were used as practice trials.

We used a 2 × 3 design for correctly bisected triplets (requiring “Yes” responses) as well as incorrectly bisected triplets (requiring “No” responses). For correctly bisected triplets, the factors *multiplicativity* (yes: e.g., 21_24_27 vs. no: 22_25_28) and *lateralization* (i.e., right vs. left vs. bilateral) were manipulated. For incorrectly bisected triplets, *bisection possibility* (bisectable: e.g., 21_22_27 vs. non-bisectable: e.g., 23_26_30) and again *lateralization* (i.e., right vs. left vs. bilateral) was varied. A comprehensive description of stimulus can be found in Moeller et al., 2009).

Participants had to decide whether the triplet’s central number represented the arithmetic mean of the two outer numbers. They were required to indicate their decision by pressing either the left (i.e., “No response”) or the right response button (i.e., “Yes” response). The experiment allowed participants to take a self-defined break after 50 trials each.

#### Number Comparison Task

For the number comparison task, a subset of triplets from the study by Moeller et al. (2009) was used, whereby only the two outer numbers were offered as duplets to be compared (e.g., 22_38). This subset included 75 item pairs,^1^ 25 within-decade items with two numbers within the same decade (e.g., 22_28). The remaining 50 items were manipulated for unit-decade compatibility (i.e., compatible vs. incompatible trials). As Ratinckx et al. (2006) only observed an effect of lateralized stimuli presentation on the compatibility of the presented number pairs, we focused on the manipulation of this factor to reduce the number of items and thus total testing time. The same item set was used for each condition (i.e., right, left, and bilateral), resulting in a total of 225 number comparison tasks. All items were presented in pseudo-randomized order in one run. The same practice trials were used as in the NBT but without presentation of the central number of the triplet. Participants had to decide whether the upper number was larger than the lower number on the display by pressing either the left (i.e., “No” response) or the right response button (i.e., “Yes” response). Appendix Table A1 provides an overview of the stimulus set for the number magnitude comparison tasks.

#### Control Task

In the control task, participants’ multiplication performance was assessed to consider it in the subsequent analyses. Therefore, we used the same verification paradigm and experimental setup as used by Clemens et al. (2013). One hundred and eighty simple multiplication problems (90 with a correct and 90 with an incorrect solution probe), covering the operand range from 0 to 10, were presented in Arabic format (e.g., 7 × 5 = 35). Multiplication problems included standard problems (96), rule problems (72, with 24 problems using 0, 1, and 10 as multiplicand, respectively), and tie problems (12). In addition, 30 different multiplication problems (15 with a correct and 15 with an incorrect solution probe) were used as practice trials. Participants had to decide whether the presented solution probe of the multiplication problem was correct (i.e., “Yes” response) or incorrect (i.e., “No”- response). Appendix Table A2 in the Appendix provides an overview of the stimulus set for the control tasks.

### Apparatus, Experimental Paradigms, and Stimulus Presentation

For all experiments, a 17″ screen driven at a resolution of 1920 × 1200 pixels and 60 Hz refreshing rate was used. Participants were seated at a distance of 60 cm from the screen. Constant viewing distance was ensured by using a head and chin rest. During the experiment, the experimenter was sitting directly opposite to the participant to control eye fixation. In case of a loss of fixation, the experimenter reminded the participant to fixate the center of the screen, which was only necessary in a few trials in 6 of 23 participants. Participants gave their answers on a standard QWERTZ keyboard with the keys necessary for the experiments labeled. In-ear headphones for the examiner were connected to the laptop so that an acoustic signal, which only the examiner could hear, could be used to indicate the beginning of each trial.

In the divided visual field paradigm, experimental setup and stimulus presentation for the experimental tasks (i.e., number comparison and NBT) was similar to the study of Ratinckx et al. (2006). Items were presented tachistoscopically in four different visual hemifield displays, this means, two unilateral conditions (right and left) and two bilateral conditions. For the bilateral conditions, the item set was split in half so that numbers in the first half of items were displayed in the upper left and lower right corner, respectively (i.e., bilateral A), and numbers in the second half of items were presented in the lower left and upper right corner (i.e., bilateral-B). Bilateral condition A and B were collapsed for subsequent data analysis. In the experimental design of Ratinckx et al. (2006), however, visual input differed between the unilateral and bilateral condition as follows: In the unilateral condition, target items were presented either to the left or to the right of a centrally presented fixation cross. In the bilateral condition, target items were presented to the left and right of the fixation cross while empty positions were filled “##.”

**FIGURE 1:**
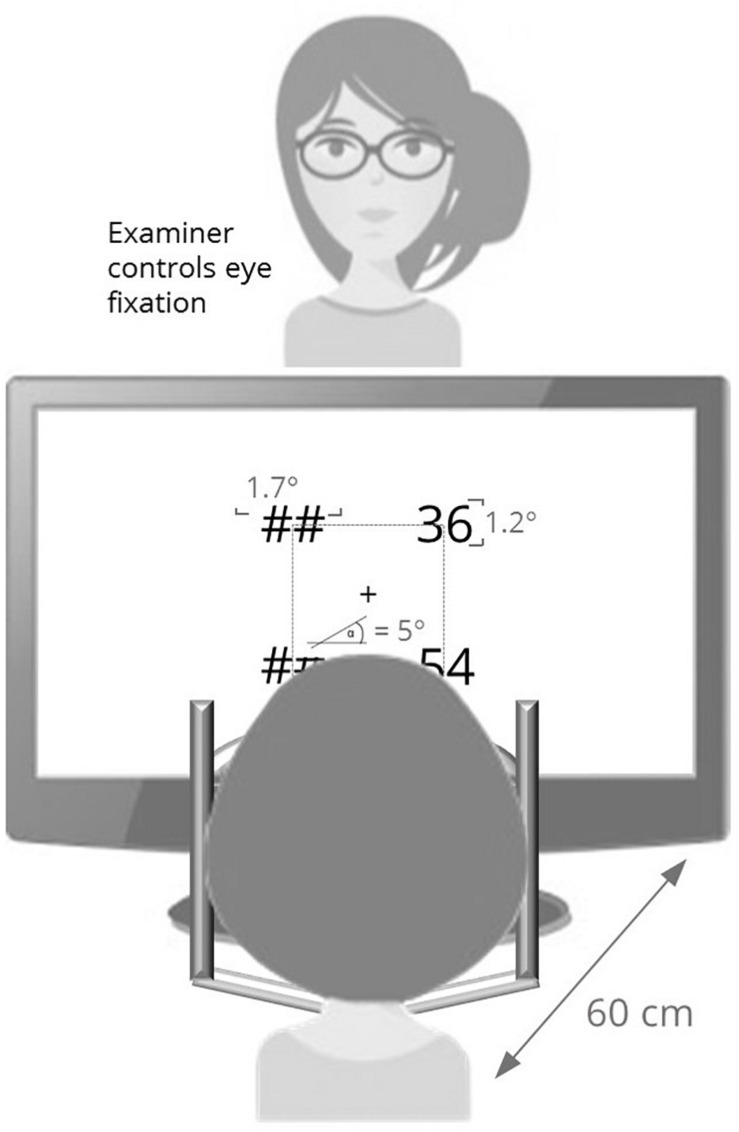
Experimental setup for the lateralized stimulus presentation.

In the present study, we aimed at balancing perceptual load across unilateral and bilateral presentation of stimuli. Thefore, we adapted the experimental paradigm slightly (cf. Figure 1): In the center of the screen, a fixation cross (i.e., plus [+] sign, font: Arial, size: 30), which extended 0.5° of visual angle horizontally and vertically, was presented. An imaginary square measuring 5 °–5 ° (cf. Ratinckx et al., 2006; for the trigonometrical constraints of the bilateral condition) was centered around the fixation stimulus. The diagonal projection between fixation location and corners of the imaginary square followed a 45° angle.

**FIGURE 2:**
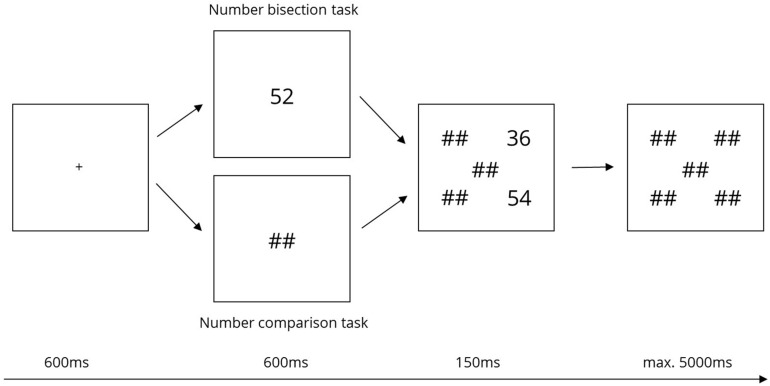
Trial sequence for the NBT (top) and number comparison task (bottom).

Two-digit Arabic numbers ranging from 11 to 99 were presented (extending 1.7° of visual angle horizontally and 1.2° vertically) at the corners of the imaginary square centered around the fixation cross. At corners not occupied by numerical stimuli, “##” was presented to keep visual input comparable across conditions (see Figure 2). For example, in the right unilateral presentation condition, numbers were displayed to the right of the fixation cross and “##” were displayed left from the fixation cross. Figure 1 provides an overview of the experimental setup. Due to this setup, conditions were presented in a randomized order (cf. Ratinckx et al., 2006, for conditions presented block-wise).

At the beginning of each trial, the fixation cross was displayed for 600 ms. Simultaneously, an acoustic signal was presented to the experimenter through the headphones. This acoustic signal indicated the beginning of a new trial to the experimenter because he/she was unable to see the screen from his/her position but had to monitor eye-movements of the participants. In the case of the NBT, the central number of the upcoming triplet was presented at the position of the fixation cross for another 600 ms. Then, “##” replaced the central number and the triplet’s outer numbers were tachistoscopically presented for 150 ms at two corners of the imaginary square. Finally, all positions were covered by “##” for a maximum of 3650 ms or until a response key was pressed. In total, one trial lasted up to 5000 ms. Figure 2 illustrates the trial sequencing. In experimental trials, participants were instructed to fixate the fixation sign throughout the entire trial and not to move their eyes.

In the control task, items were not presented in a divided field paradigm. Instead, we used the same experimental setup as Clemens et al. (2013). In this setup, the overall presentation time for each multiplication problem was variable. The multiplication problem disappeared immediately after the response was given with a maximum presentation time of 3000 ms. Each multiplication problem was followed by a mask (“######”) presented for 500 ms, to keep trials separated from each other.

### Data Analysis

Data were analyzed using the open source language and statistical environment R (Version 3.6.; R Core Team, 2019). All analyses were done on the rate correct score (RCS; Woltz and Was, 2006), a combined speed (RT) and accuracy measure of performance. The RCS was calculated by combining the proportion of correctly solved trials and average RT for each condition (i.e., lateralization, multiplicativity, compatibility, etc.) to make the measure comparable across conditions (cf. Vandierendonck, 2017) to reflect the number of correct answers per second. Participants were excluded from data analysis when they scored less than 60% correct in one of the tasks. Only RTs for correct responses (both “Yes” and “No” answers) larger than 200 ms in the number bisection task and 150 ms in the number comparison task were analyzed. Incorrect or missing responses were not considered.

In the NBT, effects of multiplicativity, bisection possibility and lateralization on the RCS were evaluated separately for correctly bisected and incorrectly bisected triplets. Additionally, the effect of multiplicativity was controlled for individual multiplication performance assessed by the control task. In the number comparison task, we analyzed influences of compatibility and lateralization on the RCS. Prior to this, we compared participants’ performance (i.e., mean percentage correct and mean RT) in our study to participants’ performance in the study by Ratinckx et al. (2006).

## Results

In total, complete data sets of 23 participants entered analyses (6 male, mean age: 24.34, *SD* = 3.03). Eight participants had to be excluded for scoring below 60% correct in the NBT. All of these participants had more than 10 years of formal education. Mean LQ was 84.53 (*SD* = 16.01) according to the Edinburgh handedness inventory. Eye dominance was defined as predominantly right for the distance (right = 14 participants, left = 9 participants). Table 1 summarizes the results of the two experimental tasks and the control task (i.e., percentage correct, reactions times, and the RCS).

**TABLE 1:** Overview of the mean test performance in the different tasks providing mean percentage correct sores, mean reaction times and the mean rate correct score (RCS) for the different tasks.

	**Right**		**Left**		**Bilateral**	
	**Mean**	***SD***	**Mean**	***SD***	**Mean**	***SD***
**Number bisection task**						
*Multiplicative triplets*						
Percentage correct	61.56	13.90	62.00	13.65	64.17	13.33
Reaction time	1666.52	447.04	1668.87	443.43	1758.54	467.15
Rate correct score (RCS)	0.40	0.15	0.41	0.16	0.39	0.15
*Non-multiplicative triplets*						
Percentage correct	37.39	7.22	39.96	7.08	37.13	5.52
Reaction time	1470.50	385.38	1515.72	407.71	1566.29	405.71
Rate correct score (RCS)	0.28	0.10	0.26	0.09	0.24	0.08
*Bisectable triplets*						
Percentage correct	72.87	9.99	66.87	10.82	69.72	11.66
Reaction time	1607.36	420.62	1603.21	381.89	1695.61	374.00
Rate correct score (RCS)	0.48	0.14	0.43	0.12	0.43	0.12
*Non-bisectable triplets*						
Percentage correct	75.72	11.04	72.60	12.68	76.60	11.64
Reaction time	1614.37	392.82	1594.32	396.56	1665.54	354.88
Rate correct score (RCS)	0.49	0.11	0.48	0.14	0.48	0.12
**Number bisection task**						
*Compatible pairs*						
Percentage correct	85.73	5.63	83.82	5.04	84.87	8.44
Reaction time	919.49	170.65	921.00	200.65	919.10	191.94
Rate correct score (RCS)	1.20	0.39	1.00	0.22	0.90	0.17
*Incompatible pairs*						
Percentage correct	77.04	12.42	81.91	12.17	83.82	11.78
Reaction time	908.74	173.48	910.33	204.68	944.12	167.68
Rate correct score (RCS)	0.89	0.21	0.74	0.15	1.04	0.32
**Control task**						
Percentage correct	–	–	–	–	90.50	4.65
Reaction time	–	–	–	–	1248.09	215.8
Rate correct score (RCS)	–	–	–	–	0.75	0.15

### Number Bisection Task

First, correlating reaction times and percentage correct trials revealed a relatively high correlation (Spearman correlation: *r_*s*_* = 0.49). This correlation suggests the presence of a speed-accuracy trade-off in solving the NBT and, thus, may warrant the use of the RCS in subsequent analyses.

**FIGURE 3:**
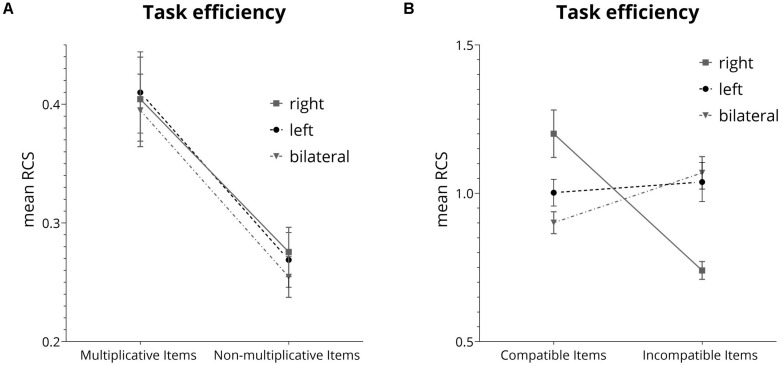
**(A)** Effect of multiplicativity for the mean rate correct score (RCS). **(B)** Interaction between lateralization conditions and unit-decade compatibility calculated for the mean rate correct score (RCS).

For correctly bisected triplets, we evaluated modulations of the multiplicativity effect using a 2 (multiplication: multiplicative triples vs. non-multiplicative triples) × 3 (lateralization: right vs. left vs. bilateral) analysis of covariance (ANCOVA) with the RCS as dependent variable. There was a significant main effect of multiplicativity [*F*(1,131) = 47.08, *p* < 0.001, η^2^*_*p*_* = 0.23)] prevailing after controlling for test performance in multiplication facts [*F*(1,131) = 29.79, *p* < 0.001, η^2^*_*p*_* = 0.14)]. This indicated that triplets which are part of multiplication tables were responded to with more correct responses per second as compared to triplets not part of multiplication tables (see Figure 3A). The main effect for lateralization was not significant [*F*(2,131) = 0.19, *p* = 0.82, η^2^*_*p*_* = 0.0018], neither was the interaction [*F*(2,131) = 0.058, *p* = *0.94*, η^2^*_*p*_* < 0.001].

Additionally, we conducted a *post hoc* analysis in order to further investigate the null effect of lateralization on multiplicative items in the NBT. To this end, we included the problem size in our *post hoc* analysis by conducting a median split. We considered large problem sizes to be those where the smallest of a triplet’s numbers was greater than 46. When our items were less reflective of fact knowledge and required more cognitive demand, multiplicativity should play a smaller role in larger problem sizes. In other words, triplets like 18_27_36 would be more closely associated with being dividable by 9 than triplets like 63_72_81. Therefore, we ran a multiple linear regression predicting RCS based on multiplicativity and problem size of items to check this hypothesis.

A significant regression equation was found with an *R*^2^ of 0.66 [*F*(3, 260) = 172.2, *p* < 0.001]. Multiplicativity significantly increased participant’s RCS. Problem size instead was not a significant predictor of the RCS. However, the interaction of multiplicativity and problem size was significant with *p* < 0.001, indicating that multiplicativity of triplets was specifically beneficial when triplets consisted of smaller numbers. Results are displayed in Table 2.

**TABLE 2:** Linear regression to check the influence of problem size.

**Variable**	**Estimate**	***SE***	***t*-Statistic**	***p*-Value**
Intercept	8.595e−05	1.767e−05	4.86	<0.001
Multiplicativity^*a*^	4.941e−04	2.500e−05	19.78	<0.001
Problem size^*a*^	2.658e−05	2.500e−05	1.06	0.29
Multiplicativity × Problem size	−2.513e−04	3.535e−05	−7.11	<0.001

*Adjusted *R^2^* = 0.66; ^*a*^Factors “Multiplicativity” and “Problem size” have been dummy coded (1 = multiplicative and 1 = large problem size).*

For incorrectly bisected triplets, we evaluated the effect of bisection possibility and lateralization using a 2 (bisection possibility: bisectable vs. non-bisectable triples) × 3 (lateralization: right vs. left vs. bilateral) analysis of variance (ANOVA) with the RCS as the dependent variable. There was no significant main effect for bisection possibility [*F*(1,132) = 2.16, *p* = 0.14, η^2^*_*p*_* = 0.002], indicating that task efficiency did not differ between bisectable (*M* = 0.45, *SD* = 0.02) and non-bisectable triplets (*M* = 0.48, *SD* = 0.11). Moreover, there was no main effect for lateralization [*F*(2,132) = 0.87, *p* = 0.42, η^2^*_*p*_* = 0.012] nor a significant interaction [*F*(2,132) = 0.36, *p* < 0.69, η^2^*_*p*_* = 0.005].

### Number Comparison Task

**FIGURE 4:**
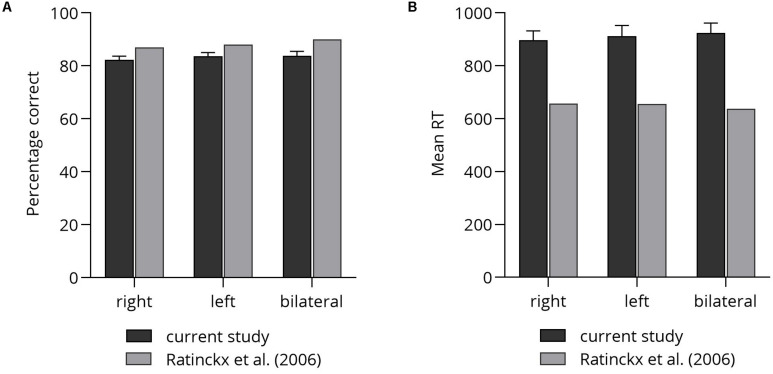
Comparison of participant’s test performance: **(A)** mean percentage correct. **(B)** Mean RT. Error bars depict SEM.

First, in view of the rather poor performance of participants in the NBT, we checked whether participants’ performance (i.e., mean percentage correct and mean RT) in the number comparison task in the current study was comparable to participants’ performance reported by Ratinckx et al. (2006). Figures 4A,B illustrates participants’ performance in both studies.

Independent-samples *t*-tests were conducted, one for each lateralization condition (i.e., right, left, bilateral), separately for correctness and reaction times. *p*-Values were corrected for multiple applying the procedure suggested by Bonferroni. In terms of correctness, significant differences were observed between lateralization conditions in the present study (right: *M* = 82.31, *SD* = 6.68, left: *M* = 83.71, *SD* = 6.53, bilateral: *M* = 83.82, *SD* = 7.92) and the study by Ratinckx et al. (2006; right: *M* = 87, left: *M* = 88, bilateral: *M* = 90), *t_*m*__*in*_*(22) = 3.37, *p* < 0.001. Despite the significant differences, performance differed by no more than seven percentage errors. Interestingly, correctness in both studies was highest in the bilateral condition and lowest in the right lateralized condition.

In terms of reaction times, participants in the current study showed on average longer reaction times (right: *M* = 896 ms, *SD* = 171, left: *M* = 912 ms, *SD* = 189, bilateral: *M* = 924 ms, *SD* = 178.46) than did participants reported by Ratinckx and colleagues (right: *M* = 658 ms, left: *M* = 656 ms, bilateral: *M* = 637 ms), *t*_*min*_(22) = 7.73, *p* < 0.001 – suggesting higher task demands in the current study.

Second, influences of lateralized presentation of stimuli on the unit-decade compatibility effect were evaluated by running 2 (compatibility: compatible vs. incompatible pairs) × 3 (lateralization: right vs. left vs. bilateral) ANOVA on the RCS as the dependent variable. There was a marginally significant main effect of compatibility [*F*(1,132) = 3.51, *p* = 0.06, η^2^*_*p*_* = 0.02], indicating more correctly solved items per second for compatible (*M* = 1.06, *SD* = 0.28) as compared to incompatible number pairs (*M* = 0.93, *SD* = 0.27). The main effect for lateralization was not significant [*F*(2,132) = 0.46, *p* = 0.66, η^2^*_*p*_* = 0.0004]. However, the interaction of compatibility and lateralization was significant [*F*(2,132) = 17.78, *p* < 0.001, η^2^*_*p*_* = 0.21, illustrated in Figure 3B]. The same interaction was found in the study by Ratinckx et al. (2006).

*Post hoc* comparisons for lateralization using Tukey HSD controlling for multiple comparisons showed a significant [*p* = 0.002] difference between presentation to the right visual hemifield (*M* = 1.20, *SD* = 0.39) and bilateral presentation (*M* = 0.90, *SD* = 0.17) for compatible number pairs. Differences between these two conditions and presentation to the left visual hemifield (*M* = 1.00, *SD* = 0.22) were not significant. For incompatible number pairs, significant differences were observed between presentation to the right hemifield condition (*M* = 0.74, *SD* = 0.14) and both presentation to the left hemifield (*M* = 1.04, *SD* = 0.23; *p* = 0.003) and the bilateral presentation condition (*M* = 1.07, *SD* = 0.26, *p* < 0.001). Furthermore, compatible trials (*M* = 1.20, *SD* = 0.39) differed significantly [*p* < 0.001] from incompatible trials (*M* = 0.74, *SD* = 0.14) only in the right visual hemifield field condition.^2^

## Discussion

The present study aimed at evaluating the postulate of the TCM that the verbal representation of arithmetic facts should be situated unilaterally in the left hemisphere of the human brain (Dehaene and Cohen, 1995, 1997). Therefore, we investigated whether processing of multiplicative triplets in the NBT shows a significant disadvantage when visual input is transmitted to the contralateral right hemisphere only using a divided visual field paradigm. To ensure applicability of the divided visual field paradigm, participants also completed a magnitude comparison task for which influences of lateralized presentation of stimuli was observed previously (Ratinckx et al., 2006).

As regards the latter, we replicated the results by Ratinckx et al. (2006), in particular the modulation of the unit-decade compatibility effect by the lateralization of input presentation in the divided visual field paradigm: presentation of the numbers in the right hemifield (and thus transmitted to the left hemisphere) increased the disadvantage for incompatible number pairs when comparing their magnitude in contrast to the presentation of number pairs in the left visual hemifield or bilaterally.

As regards the NBT, we replicated both standard numerical effects, this means the multiplicativity effect and the bisection possibility effect (e.g., Nuerk et al., 2002; Wood et al., 2008; Moeller et al., 2009, 2011). However, contrary to the hypothesis deriving from the TCM, we did not observe modulation of the multiplicativity effect by lateralization of stimulus presentation. In the following, we will discuss these findings in more detail step by step.

### Unit-Decade Compatibility Effect in Number Magnitude Comparison

In line with the results of Ratinckx et al. (2006), no particular disadvantage of processing the more difficult incompatible number pairs items was found when stimuli were presented bilaterally or within the left hemifield. Bilateral presentation in both visual fields and unilateral presentation in the left visual hemifield allowed for direct processing of the respective stimuli in the right hemisphere, where the integration of tens and units into the place-value structure of the Arabic number system was argued to take place (Wood et al., 2008). The replication of the results by Ratinckx et al. (2006) suggests that our experimental setting was principally valid for detecting differences caused by the lateralized presentation of stimuli. On a more basic level, these results indicate that participants were able to perceive, and process the presented two-digit numbers even though these were presented only briefly in perifoveal position.

Nevertheless, it has to be noted that participants in our study committed more errors and took longer for their responses as compared to the participants in Ratinckx et al. (2006). While we cannot rule out that this difference might in part be due to unspecific individual differences or cultural differences between participant samples tested (e.g., Dutch vs. German undergraduate students), there were also differences in the experimental setting, which might have contributed to differences in overall accuracy and reaction times of results.

First, Ratinckx et al. (2006) realized lateralization condition block-wise so that within one block all stimuli were presented in the same visual hemifield only. This way, participants knew where to expect the perifoveally presented stimuli (i.e., right, left or bilaterally). By presenting stimuli in different visual hemifields in randomized order, we aimed at preventing attentional orientation toward the left or the right side before the actual stimuli were presented. However, randomized order of stimuli might also have led to longer reaction times and higher errors rates because participants might have experienced more difficulties in locating and perceiving the stimuli.

Second, we presented visual input in all four locations where a stimulus could potentially be presented (i.e., at each location either one of the two numbers or “##” as a mask was presented). Again, this might have increased task difficulty as more visual input needed to be processed (Poole and Kane, 2009). However, it has to be noted that this was true for all conditions. Thus, it might have potentially affected overall accuracy and reaction time in all conditions to a similar extent, while the differential pattern between lateralized presentation conditions should not have been altered. The latter is reflected by the differential results for the unit-decade compatibility effect depending on lateralization of input presentation.

In sum, we were able to replicate the differential result pattern for the unit-decade compatibility effect as reported by Ratinckx et al. (2006), suggesting that participants were able to perceive and process the two-digit numbers and, more importantly, that our experimental setting was, in principal, valid for detecting differences to due lateralized presentation of numerical stimuli.

### Multiplicativity in the Number Bisection Task

Unexpectedly, we did not observe significant modulation of the multiplicativity effect by lateralization of stimulus presentation in the NBT. Generally, this finding allows for two possible conclusions:

First, the assumption of the TCM is wrong, namely that the verbal representation, which underlies arithmetic fact retrieval such as overlearned multiplication facts, is subserved in a lateralized manner in the left hemisphere of the human brain only (cf. Dehaene and Cohen, 1995, 1997; Dehaene et al., 2003). However, before such an interpretation can be considered, other possible explanations need to be ruled out and this finding should be replicated in the same task but also in other tasks drawing on the verbal representation.

Currently, we can only state that multiplicativity as measured in the NBT was not modulated by the site of lateralized presentation in the present experiment. There may be the following reason for this observation: while multiplicativity in the NBT with two-digit number pairs has been argued to draw heavily on verbally mediated arithmetic fact retrieval (cf. Nuerk et al., 2002 for behavioral data; Wood et al., 2008 for neuroimaging data; Klein et al., 2016 for connectivity data), the effect probably may not reflect retrieval of overlearned arithmetic facts only. For instance, whenever participants operate on two-digit numbers, additional processes such as place-value integration (Nuerk et al., 2011) or working-memory (Kong et al., 2005) may be required. In line with this argument, we observed problem size to interact with multiplicativity in the NBT. The interaction specifically indicated that the processing advantage for multiplicative items was smaller for triplets with larger problem size. In turn, this indicates that multiplicativity of triplets was specifically beneficial when triplets consisted of smaller numbers. Such a problem size effect has been reported previously for both children and adults in multiplication (Campbell and Graham, 1985; De Brauwer et al., 2006; but see Domahs et al., 2006). The effect is also in line with the results of Wood et al. (2008) who showed increasing retrieval-specific activation of the left angular gyrus with decreasing problem size of multiplicative triplets in the same version of the NBT as used in the current study. This activation has been repeatedly interpreted to indicate arithmetic fact retrieval (e.g., Dehaene et al., 2003; Delazer et al., 2003; Ischebeck et al., 2006; Grabner et al., 2009). However, as outlined above our experimental setting might have been more difficult than in the study by Wood et al. (2008) due to the brief lateralized perifoveal presentation. When we also take into account the observed differences in overall behavioral performance in magnitude comparison between the study by Ratinckx et al. (2006) and the present study, this possibility can hardly been ruled out. Our way of presenting the NBT might have led to additional demands as compared to previous variants of the NBT in which all numbers were presented simultaneously and in one line. This leads us to the second possible conclusion drawn from our results.

Second, it might be the case that the NBT in its present variant was very and maybe even too difficult for participants. An indicator for this assumption seems the high error rates observed for the NBT as well as the high number of exclusions of participants due to poor performance in the NBT. In particular, from 31 participants, 8 participants had to be excluded for overall scoring below 60% correct in the NBT (with 50% being guessing rate). Additionally, this was combined with a specific pattern of significant better accuracy with slower reaction times in multiplicative triplets. Possibly, the two numbers presented laterally from the fixation cross were perceived and, due to their short presentation duration of only 150 ms, repeated in verbal working memory before a decision on the bisectability of a triplet was made. As multiplicative triplets have been shown to be processed in a verbal code (Moeller et al., 2011), decisions on the multiplicativity of these rehearsed numbers might have been more accurate, as the significant lower error rate for multiplicative triplets may indicate. This would be in line with the idea of better performance in multiplicative triplets in terms of accuracy, while, at the same time, rehearsing three numbers in verbal working memory, would be relatively slow. Support for this assumption also comes from the observation that this specific pattern of higher accuracy synced with slower reaction times was not observed for incorrectly bisected triplets. Thus, the observed behavioral pattern most probably reflects a specific facilitation of the task for multiplicative items as both the multiplication facts as well as verbal working memory operate on a phonological code (Moeller et al., 2011).

While, all these processes are assumed to be subserved by the left hemisphere (Dehaene et al., 2003), we have to consider that we assessed healthy participants with intact interhemispheric connections. Therefore, it will only take a few milliseconds until stimuli may be processed in both hemispheres due to interhemispheric connections via transcallosal fiber pathways (e.g., Chaumillon et al., 2018), so that no temporal processing advantage due to multiplicativity may be observable any more. In other words, the longer processing of the respective stimuli takes, the less likely differences due to lateralized processing in terms of speed should be observed. Therefore, we would suggest that an effect of lateralization of presentation may primarily be expected for early bottom-up effects such as the unit-decade compatibility effect when the task is easy enough. In our magnitude comparison task, only two of the briefly and lateralized presented two-digit numbers were relevant, while participants had to consider three two-digit numbers in the NBT.

In addition, and when interpreting these results, there are two constraints that need to be considered. First, saccadic eye movements were not controlled by eye-tracking; an examiner sitting opposite of the participant monitored eye fixation. Since the center of the lateralized stimuli was located 5 degrees from central fixation and was thus well beyond the critical distance of saccade amplitude that can be detected with the naked eye, we can be very sure that the current procedure has prevented unwanted loss of fixation. Nevertheless, eye-tracking could have been more precise in excluding the possibility that hemispheric asymmetries were only detected when interacting with unit-decade-incompatibility in the number comparison task but not as a main effect of lateralized processing. Second, despite our *a priori* estimation of the necessary sample size to detect hemispheric asymmetry (*N* = 21, a partial eta square of η^2^*_*p*_* = 0.20 and a power of 0.95), the sample size in the present study (*N* = 23) might have been too small to reveal a main effect of lateralized processing in both the NBT and the number comparison task. However, the observed significant modulation of the compatibility effect by lateralization of stimulus presentation suggests that hemispheric differences are present at least in the magnitude comparison task.

Therefore, it would be desirable for future studies addressing the question of lateralized processing of arithmetic fact retrieval to recruit a larger sample and use easier stimulus material (e.g., one-digit numbers). Moreover, a block-wise realization of lateralized presentation should be applied (e.g., unilateral presentation in the left-hemifield only) in a task which specifically addresses the verbal representation and retrieval of arithmetic facts such as, for instance, one-digit × one-digit multiplications.”

Finally, future studies might also evaluate possible influences of lateralized processing in brain areas that cannot be considered independently from lateralized cognitive processing. For instance, the cerebellum has been shown to indirectly regulate activation and inhibition levels of attentional networks (Mannarelli et al., 2019).

## Conclusion

The current study aimed at investigating whether the verbal representation of arithmetic facts is situated unilaterally in the left hemisphere of the human brain. While we were able to replicate both the multiplicativity effect and the effect of bisection possibility, lateralized presentation did not modulate the effect of multiplicativity.

We suggest that participants might have kept the three two-digit numbers in verbal working memory after perceiving them due to short presentation duration. This would be in line with the observed better performance in multiplicative triplets in terms of accuracy, while, at the same time, reaction times were larger. Rehearsal of the three numbers in the phonological loop was probably too time-consuming to detect fine-grained hemispheric processing asymmetries in multiplicative items due to interhemispheric connectivity. We suggest that an effect of presentation lateralization can only to be expected for early effects such as the unit-decade compatibility effect when the task is easy enough.

## Data Availability Statement

All datasets generated for this study are included in the article/Supplementary Material.

## Ethics Statement

The studies involving human participants were reviewed and approved by the Ethics Committee at the Medical Faculty of the Eberhard Karls University and at the University Hospital Tübingen (082/2018BO2). The patients/participants provided their written informed consent to participate in this study.

## Author Contributions

SJ, KM, H-OK, and EK designed the study. SJ conducted the experiment. SJ and EK analyzed the data and wrote the manuscript. SJ, KM, H-OK, and EK reviewed and approved the final version of the manuscript.

## Conflict of Interest

The authors declare that the research was conducted in the absence of any commercial or financial relationships that could be construed as a potential conflict of interest.

## Footnotes

1 Out of the 75 item pairs, 74 are taken from the triplets by Moeller et al. (2009) and one additional item was generated.

2 “Due to the contradictory findings in the literature on the influence of sex on hemispheric asymmetry, in particular for numerical processing (cf. Harris et al., 2018; Pletzer et al., 2019), we have *post hoc* analyzed the data for a potential gender effect. For this analysis, we evaluated influences of lateralized presentation of stimuli on the unit-decade compatibility effect by running a 2 (compatibility: compatible vs. incompatible pairs) × 3 (lateralization: right vs. left vs. bilateral) ANCOVA on the RCS with sex as covariate. The influence of the covariate indicated neither a significant main effect of sex [*F*(1,126) = 2.05, *p* = 0.15, η^2^*_*p*_* = 0.02] nor an interaction of sex and laterality [*F*(2,126) = 0.03, *p* = 0.96, η^2^*_*p*_* < 0.001] as well as an interaction of sex and unit-decade compatibility, respectively [*F*(,1,126) = 0.22, *p* = 0.64, η^2^*_*p*_* = 0.002]. As in the original analysis, there was a marginally significant main effect of compatibility [*F*(1,126) = 3.43, *p* = 0.06, η^2^*_*p*_* = 0.027], indicating more correctly solved items per second for compatible (*M* = 1.06, *SD* = 0.28) as compared to incompatible number pairs (*M* = 0.93, *SD* = 0.27). The main effect for lateralization again was not significant [*F*(2,126) = 0.39, *p* = 0.67, η^2^*_*p*_* = 0.0063]. However, the interaction of compatibility and lateralization was again significant [*F*(2,126) = 17.41, *p* < 0.001, η^2^*_*p*_* = 0.22].

## Appendix

**TABLE A1:** Overview of the 75 stimuli duplets in the number comparison task.

**Within decades**	**Compatible trials**	**Incompatible trials**
10_16	10_21	17_23
10_14	12_30	16_34
20_29	15_27	22_30
20_24	21_35	24_40
21_27	22_38	25_42
22_26	23_36	27_45
30_39	33_44	34_40
30_36	36_48	38_54
33_39	43_55	39_43
40_45	43_57	39_51
40_49	44_57	44_52
40_46	46_57	45_63
50_56	47_59	48_61
51_57	51_68	52_60
52_58	54_69	58_72
53_57	56_68	59_68
60_65	82_98	64_73
63_69	50_64	68_76
64_69	51_62	72_90
70_76	52_64	74_92
72_78	54_66	76_82
73_79	62_76	77_83
80_88	70_86	87_94
90_99	80_94	63_81
92_98	84_98	69_82
		

**TABLE A2:** Overview of the 180 multiplication problems for the control task.

**Correct problems**			**Incorrect problems**		
0 × 2 = 0	4 × 1 = 4	7 × 9 = 63	0 × 2 = 2	4 × 2 = 6	8 × 0 = 8
0 × 3 = 0	4 × 2 = 8	8 × 0 = 0	0 × 3 = 3	4 × 3 = 16	8 × 1 = 7
0 × 4 = 0	4 × 3 = 12	8 × 1 = 8	0 × 4 = 4	4 × 4 = 12	8 × 2 = 8
0 × 7 = 0	4 × 4 = 16	8 × 2 = 16	0 × 7 = 7	4 × 5 = 15	8 × 3 = 21
0 × 8 = 0	4 × 5 = 20	8 × 3 = 24	0 × 8 = 8	4 × 6 = 18	8 × 4 = 28
0 × 9 = 0	4 × 6 = 24	8 × 4 = 32	0 × 9 = 9	4 × 7 = 32	8 × 6 = 42
1 × 2 = 2	4 × 7 = 28	8 × 6 = 48	1 × 2 = 1	4 × 8 = 36	8 × 7 = 48
1 × 4 = 4	4 × 8 = 32	8 × 7 = 56	1 × 4 = 3	5 × 1 = 6	8 × 8 = 56
1 × 5 = 5	5 × 1 = 5	8 × 8 = 64	1 × 5 = 4	5 × 2 = 15	8 × 9 = 64
1 × 7 = 7	5 × 2 = 10	8 × 9 = 72	1 × 7 = 6	5 × 3 = 20	9 × 0 = 9
1 × 8 = 8	5 × 3 = 15	9 × 0 = 0	1 × 8 = 9	5 × 4 = 24	9 × 1 = 18
1 × 9 = 9	5 × 4 = 20	9 × 1 = 9	1 × 9 = 0	5 × 5 = 20	9 × 2 = 27
2 × 0 = 0	5 × 5 = 25	9 × 2 = 18	2 × 0 = 2	5 × 6 = 36	9 × 3 = 24
2 × 1 = 2	5 × 6 = 30	9 × 3 = 27	2 × 1 = 3	5 × 7 = 30	2 × 4 = 12
2 × 2 = 4	5 × 7 = 35	4 × 10 = 40	2 × 2 = 2	5 × 9 = 40	4 × 10 = 30
2 × 3 = 6	5 × 9 = 45	5 × 10 = 50	2 × 3 = 4	6 × 9 = 45	5 × 10 = 45
2 × 4 = 8	6 × 9 = 54	6 × 10 = 60	2 × 5 = 8	7 × 0 = 7	6 × 10 = 50
2 × 5 = 10	7 × 0 = 0	7 × 10 = 70	2 × 6 = 6	7 × 1 = 8	7 × 10 = 77
2 × 6 = 12	7 × 1 = 7	8 × 10 = 80	3 × 4 = 9	7 × 3 = 24	8 × 10 = 72
3 × 4 = 12	7 × 3 = 21	10 × 3 = 30	3 × 5 = 12	7 × 4 = 21	10 × 3 = 20
3 × 5 = 15	7 × 4 = 28	10 × 4 = 40	3 × 7 = 28	7 × 5 = 42	10 × 4 = 50
3 × 7 = 21	7 × 5 = 35	10 × 5 = 50	3 × 8 = 32	7 × 6 = 48	10 × 5 = 41
3 × 8 = 24	7 × 6 = 42	10 × 6 = 60	3 × 9 = 36	7 × 7 = 42	10 × 6 = 54
3 × 9 = 27	7 × 7 = 49	10 × 7 = 70	4 × 0 = 4	7 × 8 = 64	10 × 7 = 60
4 × 0 = 0	7 × 8 = 56	10 × 8 = 80	4 × 1 = 5	7 × 9 = 72	10 × 8 = 90
